# Toll-like receptors 7 and 9 regulate the proliferation and differentiation of B cells in systemic lupus erythematosus

**DOI:** 10.3389/fimmu.2023.1093208

**Published:** 2023-02-15

**Authors:** Luyao Wen, Bei Zhang, Xinfeng Wu, Rongzeng Liu, Hua Fan, Lei Han, Zhibo Zhang, Xin Ma, Cong-Qiu Chu, Xiaofei Shi

**Affiliations:** ^1^ Department of Rheumatology and Immunology, The First Affiliated Hospital, and College of Clinical Medicine of Henan University of Science and Technology, Luoyang, China; ^2^ Department of Immunology, School of Basic Medical Sciences, Henan University of Science and Technology, Luoyang, China; ^3^ Office of Research & Innovation, The First Affiliated Hospital, and College of Clinical Medicine of Henan University of Science and Technology, Luoyang, China; ^4^ Division of Arthritis and Rheumatic Diseases, Oregon Health & Science University and VA Portland Health Care System, Portland, OR, United States

**Keywords:** systemic lupus erythematosus, B cells, TLR signaling, TLR7, TLR9, targeted therapies

## Abstract

Systemic lupus erythematosus (SLE) is an autoimmune illness marked by the loss of immune tolerance and the production of autoantibodies against nucleic acids and other nuclear antigens (Ags). B lymphocytes are important in the immunopathogenesis of SLE. Multiple receptors control abnormal B-cell activation in SLE patients, including intrinsic Toll-like receptors (TLRs), B-cell receptors (BCRs), and cytokine receptors. The role of TLRs, notably TLR7 and TLR9, in the pathophysiology of SLE has been extensively explored in recent years. When endogenous or exogenous nucleic acid ligands are recognized by BCRs and internalized into B cells, they bind TLR7 or TLR9 to activate related signalling pathways and thus govern the proliferation and differentiation of B cells. Surprisingly, TLR7 and TLR9 appear to play opposing roles in SLE B cells, and the interaction between them is still poorly understood. In addition, other cells can enhance TLR signalling in B cells of SLE patients by releasing cytokines that accelerate the differentiation of B cells into plasma cells. Therefore, the delineation of how TLR7 and TLR9 regulate the abnormal activation of B cells in SLE may aid the understanding of the mechanisms of SLE and provide directions for TLR-targeted therapies for SLE.

## 1 Introduction

Systemic lupus erythematosus (SLE) is a common autoimmune disease that is defined by the inappropriate activation of self-reactive T and B cells and the production of autoantibodies and immune complexes that can cause irreparable organ damage (1). The variety of SLE symptoms make both diagnosis and treatment difficult. Consequently, it is of great significance to further explore the mechanism underlying the pathogenesis of SLE.

B lymphocytes perform various functions in SLE, including autoantibody production, presentation of antigen to T cells, and activation of myeloid cells to produce various cytokines (2–4). B cells can be activated by diverse factors, including Toll-like receptors (TLRs). TLRs are pathogen pattern recognition receptors that sense conserved microbial components (5). TLRs are crucial for both the development of innate immune responses and adaptive immunity, and they are thought to bridge the innate and adaptive immune systems (6). Studies show that overactivation or impaired function of the TLRs that detect nucleic acids, TLR7 and TLR9, induces dysfunction of innate immunity and breakdown of immune tolerance, which can lead to the occurrence of autoimmunity (7, 8). Both TLR7 and TLR9 are strongly associated with interferon (IFN) production by plasmacytoid dendritic cells (pDCs) (9). In addition, they are also expressed in B cells, wherein they play crucial functions (10). Surprisingly, TLR7 and TLR9 appear to play opposing roles in SLE B cells (11). TLR7 contributes to the loss of germinal centre (GC) tolerance and drives the extrafollicular B-cell response implicated in SLE (12). Critically, TLR9 in B cells seems to protect against SLE even though it is needed to produce anti-double-stranded DNA (dsDNA) antibodies (13). In fact, TLR9 has a complex dual regulatory mechanism in B cells, which can both maintain immune tolerance and participate in autoimmunity (14). This may be due to the specific presence of signalling pathways in B cells (such as BCR, CD19 and related molecules)that play a role in these processes (14, 15). In addition, the participation of co-stimulatory molecules, pro-inflammatory factors and MyD88 (which is a key molecule downstream of TLR9) also determines the fate of B cells (16, 17). Additionally, pDCs and T cells can accelerate the progression of SLE by releasing cytokines to reinforce the sensitivity of TLR7 in B cells (18–21). These findings suggest that the onset of SLE is likely significantly influenced by dysregulation of TLR7 and TLR9 signalling in B cells. However, the findings of functional and genetic studies of TLR7 and TLR9 in SLE remain controversial.

In this review, the contribution of intracellular TLR signalling in B lymphocytes to SLE is discussed. First, we present the expression levels and functions of TLR7 and TLR9 in B cells during the development of SLE. Second, we emphasize that downstream signalling pathways connected to B cell proliferation and differentiation in SLE are activated by TLR7 and TLR9 in B cells. Third, we discuss the opposing effects of TLR7 and TLR9 in B cells and their interactions in SLE. Fourth, we discuss how pDCs and T cells accelerate the conversion of B cells into plasma cells in patients with SLE through TLR signalling. Finally, we look ahead to potential strategies for targeting TLRs to treat SLE.

## 2 Expression and function of TLR7 in SLE B cells

Earlier studies have shown that the *TLR7* gene is involved in SLE (22–24). The *TLR7* gene is located on human chromosome Xp22.3. Interestingly, unlike other genes on the X chromosome, *TLR7* is able to evade silencing in females (25). Therefore, it has a higher level of expression in monocytes, pDCs and B cells in females than in males (26). Similar to the case in humans, this female-biased increased expression of X-linked genes is also present in mice susceptible to autoimmune diseases (27). In addition, *TLR7* may be coexpressed with other X-linked genes (e.g., *CXorf21*) to jointly promote the occurrence of autoimmune diseases with gender bias (28). With the increased use of genome-wide association studies (GWAS), researchers have found that SLE risk and clinical phenotype and the production of autoantibodies in SLE are influenced by *TLR7* single-nucleotide polymorphisms (SNPs) (29, 30). This finding partly explains the heterogeneity of SLE. With the rapid development of gene sequencing technologies, TLR7 mRNA has been found to be upregulated in the peripheral blood mononuclear cells of SLE patients by using bioinformatics analysis (31). Another investigation found that the expression of TLR7 in CD19^+^ B cells in SLE patients was markedly elevated compared to that in healthy individuals (32). Another study showed that SLE patients had expansion of CD19^+^CD24^hi^CD38^hi^ transitional B cells and increased release of autoantibodies due to overexpression of TLR7 (33). Similarly, transitional T1 B cells expansion and autoantibodies production can be found when overexpression of TLR7 in transgenic mice (34). In addition, *Tlr7* transgenic mice spontaneously manifest SLE-like illnesses in a B-cell-dependent manner (35). Furthermore, Hwang et al. (36) demonstrated that upregulation of TLR7 in SLE-prone mice induces B-cell marginal zone impairment, elevated levels of antibodies targeting RNA/protein complexes in B cells, and progression of disease. Brown et al. (37) reported that a child with serious lupus had a novel *de novo* missense mutation in TLR7 (TLR7^Y264H^). The variant selectively enhances sensing of guanosine and 2’,3’-cyclic guanosine monophosphate (2’,3’-cGMP) and triggers lupus-like disease when introduced in mice (generating so-called kika mice). Further studies have found that the Age-associated B cells (ABCs) of kika mice have higher TLR7 expression than those of WT mice (but lower TLR7 expression than *Tlr7*-double-positive Yaa mice). Nevertheless, the effects of the Y264H mutation were more serious than those of mutations of the *Yaa* allele, with kika mice showing higher levels of autoantibodies (37). Moreover, when wild-type (WT) mice of diverse genetic backgrounds were treated with the TLR7 agonists imiquimod and R848 for 4 weeks, they developed systemic autoimmune disease accompanied by an increased titre of autoantibodies against nucleic acids and multiple organ damage, including glomerulonephritis, carditis, hepatitis, and photosensitivity (38). According to additional research, focusing on the TLR7 pathway in lupus-prone models may provide insights for preventing the onset of serious disease (39). The above studies show that TLR7 promotes the occurrence and development of SLE at the genetic and molecular levels. These findings suggests that TLR7 is a candidate for targeted therapy for SLE.

## 3 TLR7-mediated disease mechanisms in SLE B cells

**Figure 1:**
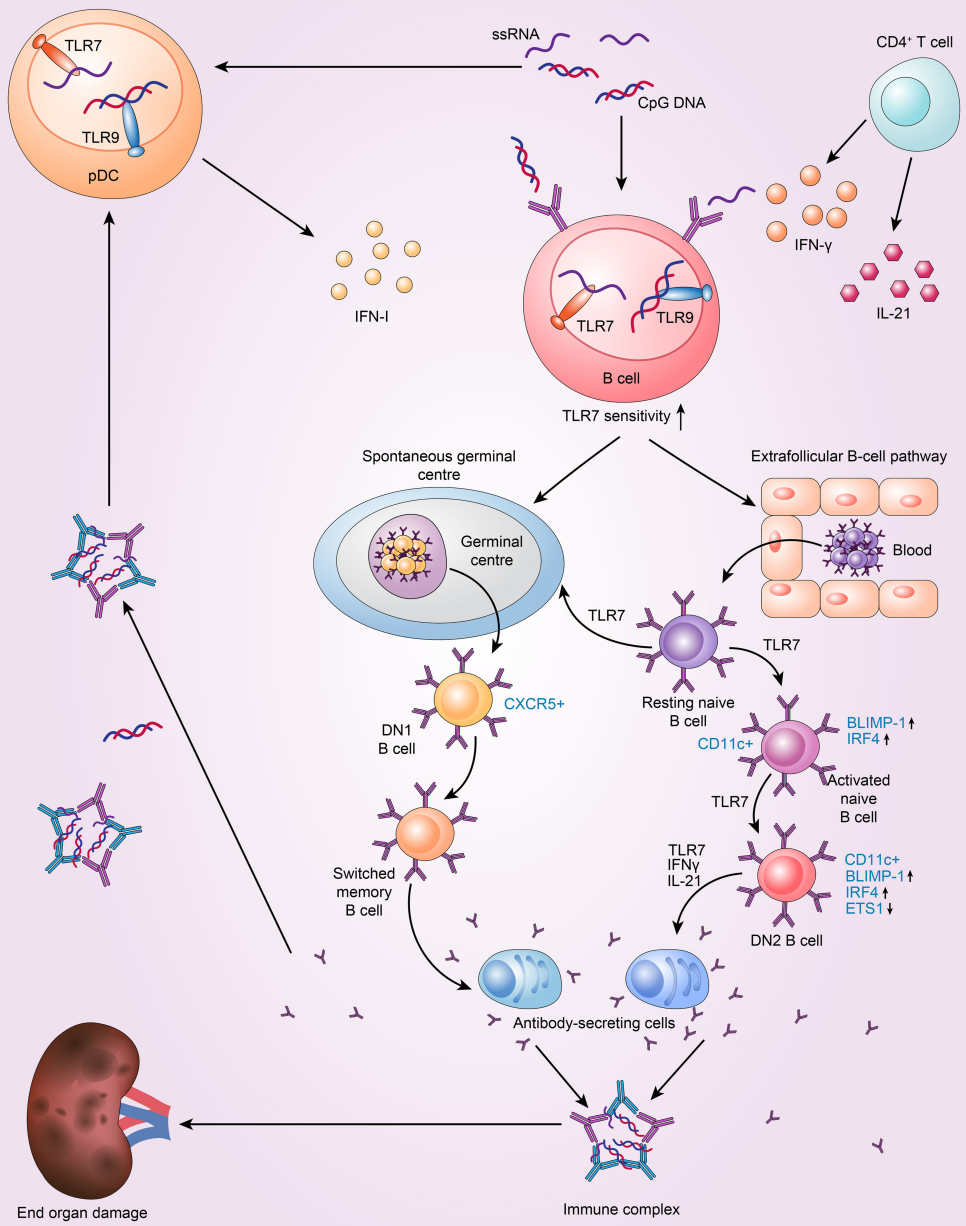
TLR7 drives the extrafollicular B-cell response and the loss of germinal centre (GC) tolerance, which is implicated in the pathogenesis of SLE. 1. Resting naive B cells enter the germinal centre pathway, which generates DN1 B cells and memory B cells, and then differentiate into antibody-secreting cells. The above processes are dependent on TLR7. 2. TLR7 drives the extrafollicular pathway, in which resting naive B cells successively become activated naive B cells, DN2 subset B cells and finally antibody-secreting cells. 3. pDC and T cells intensify TLR7 sensitivity to drive autoreactive B-cell development into antibody-secreting cells by secreting IFN-I, IFN-γ and IL-21.

TLR7 contributes to the loss of germinal centre tolerance and drives the extrafollicular B-cell response implicated in SLE **(** **Figure 1**). In the periphery, GCs are a crucial place where B-cell–tolerance is assessed (40). Studies have shown that the GC response and production of antibodies are significantly decreased in *Tlr7-*null mice B cells upon RNA virus infection (41). Consistent with these findings, TLR7 in B cells is essential for GC production in the B^WAS−/−^ model, in which B cells lack the Wiskott-Aldrich syndrome protein (42). In another mouse model with two copies of *Tlr7*, GCs fail to eliminate markedly autoreactive B cells, and these cells undergo subsequent clonal expansion and somatic mutation and then enter the plasma-cell compartment (43). This finding suggests that B-cell-intrinsic TLR7 can disrupt GC tolerance. Another study showed that B6.Sle1b mice treated with a TLR7 agonist have increased numbers of spontaneous germinal centres (Spt-GCs) (44). Conversely, *Tlr7* deficiency prevented the formation of Spt-GCs in autoimmune B6.Sle1b mice and reduced the production of autoantibodies (44). Additionally, a lack of TLR7/MyD88 impairs B-cell proliferation and survival after stimulation compared to B6 controls (44). These results demonstrate that the development of Spt-GCs and elevated autoantibody production in SLE are mediated by B-cell-intrinsic TLR7 signalling.

Furthermore, in cases of active SLE, TLR7 can promote extrafollicular B-cell differentiation, causing resting naive B cells to constantly differentiate into activated naive B cells, DN2 B cells, and ultimately Ab-secreting cells (12). Individuals with SLE have higher levels of activated naive B cells(CD11c^+^IgD^+^CD27^−^CD21^−^MTG^+^CD23^−^) and the DN2 subset of IgD^−^CD27^−^ double-negative B cells (DN2 B cells; IgD^−^CD27^−^CD11c^+^T-Bet^+^CD69^+^CD21^−^CD24^−^CD38^−^CXCR5^−^FCRL4^−^FCRL5^+^) (45). Moreover, SLE patients with increased abundance of DN2 B cells have more anti-RNA and anti-Sm/RNP autoantibodies and exhibit higher disease activity (12). DN2 cells are the predominant B-cell population in active SLE; they tend to develop into plasma cells and exhibit high levels of CD11c and T-Bet expression but low levels of CXCR5 and CD62L expression (12). Interestingly, the phenotype of DN2 cells is distinct from that of activated naive B cells. The two cell types share a similar transcriptional profile, with only 42 differentially expressed genes between them (12, 46). Furthermore, DN2 B cells are thought to be derived from activated naïve B cells (12). B lymphocyte-induced maturation protein 1 (BLIMP-1) and IFN regulatory factor 4 (IRF4) levels are higher in these two types of cells and control plasma cell differentiation and the expression of transcription factors (TFs) related to plasmablasts (47). DN2 cells express low levels of ETS proto-oncogene 1 (ETS1), a CD22-regulated TF that inhibits plasma cell differentiation (48). These features result in the accumulation of extrafollicular autoreactive B cells (49). SLE DN2 cells are hyperresponsive to TLR7 agonists, and TLR7-mediated upregulation of costimulatory molecule expression and suppression of the expression of CD72 (a receptor) inhibit the activation of endosomal TLR7 (50). Moreover, chronic TLR7 activation in lupus models is sufficient to cause CD11c^+^ B cells to differentiate and produce anti-Sm/RNP autoantibodies, whereas B-cell intrinsic TLR7 deficiency reduces the number of CD11c^+^ B cells and ameliorates lupus-like conditions (42). Indeed, TLR7 possesses stimulatory action in these autoreactive B-cell subsets and, in both humans and animals, can cause naive B-cell differentiation towards DN2 cells and plasma cells with the help of IL-21 and IFNγ (47, 51). In SLE patients, IL-21 promotes the growth of CD11c^+^T-Bet^+^CD21^−^CD38^−^B cells, which resemble DN2 B cells and are autoreactive and associated with clinical symptoms (18). Furthermore, TLR7 and B-cell receptor (BCR) agonists cooperate with IFNγ, IL-2, IL-21 and B-lymphocyte stimulator (BlyS) to stimulate naive human B cells in SLE, and an *in vitro* study reported that the produced IgD^−^CD27^−^CD11c^+^T-Bet^hi^CD21^−^CXCR5^−^IRF4^int^FcRL5^+^ B cells resemble DN2 B cells (52). Age-associated B cells (ABCs), the mouse equivalent of DN2 B cells, are another crucial B-cell subpopulation in SLE. These CD11b^+^CD11c^+^T-Bet ^+^ cells are increased in various mouse models of lupus, including *Mer* ^−/−^ and SWEF-deficient mice; these cells are also especially sensitive to TLR7 and differentiate into autoantibody-secreting cells (53–55). Another study found that ABCs in C57BL/6 mice can also present antigens to T cells (56). Specifically, the *Tlr7*-null lupus-prone mouse model lacks CD11c^+^T-Bet^+^CD21^−^ B cells. Moreover, with repeated TLR7 agonist stimulation, ABCs accumulate rapidly in mice based on a specific TLR7 signalling pathway in B cells (51). Brown et al. (37) identified that even though there was marked Spt-GC formation in *Tlr7* ^Y264H^ mice, the lupus-like symptoms did not resolve with GC formation deficiency, thus indicating that extrafollicular ABCs may be the main reason for pathogenicity (37). In summary, the GC reactions and extrafollicular responses lead to B cell activation and the formation of plasma cells *via* B-cell-intrinsic TLR7 signalling in SLE. Additionally, TLR7 signalling restricts the proliferation of B10 cells, a regulatory B-cell subpopulation that generates the immunosuppressive cytokine IL-10, in an IFNγ-dependent manner (57).

## 4 Expression and function of TLR9 in SLE B cells

TLR9 is a receptor that recognizes viral and bacterial DNA containing unmethylated cytosine–phosphate–guanine (CpG) motifs (58). One study showed that anti-dsDNA antibody levels and proteinuria in childhood-onset lupus nephritis (LN) are associated with the expression of TLR9. This result suggests that TLR9 is involved in the pathophysiology of LN (59). Consistent with these results, investigation has demonstrated that whole blood TLR9 mRNA levels are considerably greater in SLE patients than in healthy controls and are associated with the severity and type of renal pathology (60). Furthermore, the levels are positively correlated with SLEDAI grade, anti-dsDNA antibody titre, and interleukin-6 (IL-6) and other cytokine levels but negatively correlated with complement C3 levels (61). Notably, during a two-year follow-up period, patients with poor prognoses still had elevated TLR9 mRNA levels (62). Compared with the levels at SLE onset, the levels of TLR9 mRNA in patients with a favourable prognosis were obviously decreased (62). Strikingly, studies show that *Tlr9* deficiency in B cells in murine models of lupus is sufficient to accelerate renal disease even though deplete anti-nucleosome antibodies. Moreover, overexpression of B-cell–specific *Tlr9* induces remission of nephritis (13). In contrast, *Tlr9* deficiency in other cell types, including pDCs, dendritic cells, and neutrophils, has no obvious effect on LN (13). These lines of evidence suggest that TLR9 in B cells plays a specialized role in restricting the formation of autoantibodies and preventing the onset of disease. That the expression of TLR9 in SLE is proportional to disease activity appears to contradict its protective function, suggesting a unique and complex role of TLR9 in SLE B cells.

## 5 TLR9-mediated disease mechanisms in SLE B cells

Although B cells are crucial to the pathophysiology of SLE, the aberrant pathways that result in the loss of B-cell tolerance are unknown. A significant fraction of autoreactive B cells are eliminated throughout development (63), but self-reactive and polyreactive clonotypes still make up a significant portion of mature B-cell pools (40, 64). This indicates that there may be activation-associated checkpoints that can prevent these cells from differentiating into memory B cells and producing antibodies targeting self-antigens. However, patients with SLE likely have impaired peripheral and central B-cell tolerance checkpoints, leading to an accumulation of autoreactive B cells in their blood (65). TLR9 is expressed in the spleen and skeletal muscle in mammals and in peripheral blood mononuclear cells, mainly in pDCs and B cells. However, the biological effects are different in these cells. Endogenous or exogenous nonmethylated CpG-DNA can be recognized by BCR and internalized into B cells, and the process may involve phosphatidylinositol 3-kinases (PI3Ks) (66). Subsequently, the CpG-DNA is transported into early endosomes and ultimately transported into late endosomes containing TLR9, by which it is specifically identified, activating downstream signalling pathways and triggering inflammatory responses and immune effects (66). TLR9 can also promote B cell differentiation into plasma cells through germinal centre (GC) formation and extrafollicular B-cell response. However, unlike TLR7, TLR9 has a complex role in B cell differentiation and is affected by multiple elements. This results in TLR9 acting as both an immune checkpoint to maintain peripheral and central tolerance and to participate in autoimmunity (14). Rookhuizen and DeFranco discovered that TLR9-MyD88-dependent signalling in B cells increased GC output by increasing affinity maturation, the production of memory antibodies, a transition to the IgG2a subclass of antibodies and by specifically producing high-affinity antibodies (67). Additionally, the combined ligation of TLR9 and BCR has also been demonstrated to activate autoreactive B cells in previous studies (68–71). Moreover, these activated autoreactive B cells develop into extrafollicular plasma cells that can facilitate the generation of IgG2 isotype autoantibodies (72, 73). Eckl-Dorna and Batista (74) found that for both *in vitro* and *in vivo* studies, Ag-CpG conjugates are effective at promoting B cell proliferation and differentiation into short-lived extrafollicular plasma cells *via* intrinsic TLR9. In addition, ABCs do not respond to BCR ligation alone, but they quickly multiply when TLR9 is activated in aged mice (75). Activated B cells are prepared for an ABC fate by B cell-intrinsic TLR9 signals, but they also initiate a cell death program (53). Cancro’s group (14, 76) have posited that antigen taken up by the BCR is internalized and interacts with innate molecular pattern sensors. If TLR9 is activated (either alone or in conjunction with TLR7), the cell is also primed to adopt an effector or memory T-Bet^+^ ABC fate. However, the default pathway is cell cycle arrest and apoptosis, unless it is avoided by prosurvival signals or costimulation *via* cognate T cell assistance. In reality, studies have revealed that BCR can bind to TLR9 agonists on all premature murine B-cell subsets, and CD27^–^ human B cells can undergo apoptotic death after an initial proliferative burst that involves hyperphosphorylation of nuclear factor-κB (NF-κB) and p38 mitogen-activated protein kinase (MAPK) (16, 77). Moreover, p38 inhibits the persistent expansion and the survival of autoreactive B cells by inducing G1 cell cycle arrest and mitochondrial apoptosis (78, 79). However, B cells going through this stage can be saved, and the way they are saved influences what happens to them thereafter (16). In fact, impaired TLR9 function, BlyS stimulation, cognate T-cell-mediated effects or CD40 costimulation with IL-21 or IFN-γ can prolong the lifespan of autoreactive B cells (80, 81). Interestingly, a study using CpG to stimulate B cells and pDCs isolated from SLE patients who had not received hydroxychloroquine treatment found decreased expression of both B-cell activation molecules and numerous cytokines in B cells (15). However, normal reactions appear in pDCs from SLE patients; therefore, it appears that there is defective TLR9 function in SLE B cells (15). This impaired TLR9 function might prevent SLE-related autoreactive B-cell death and, consequently, harmful autoantibody generation.

## 6 TLR Signalling in B cells

**Figure 2:**
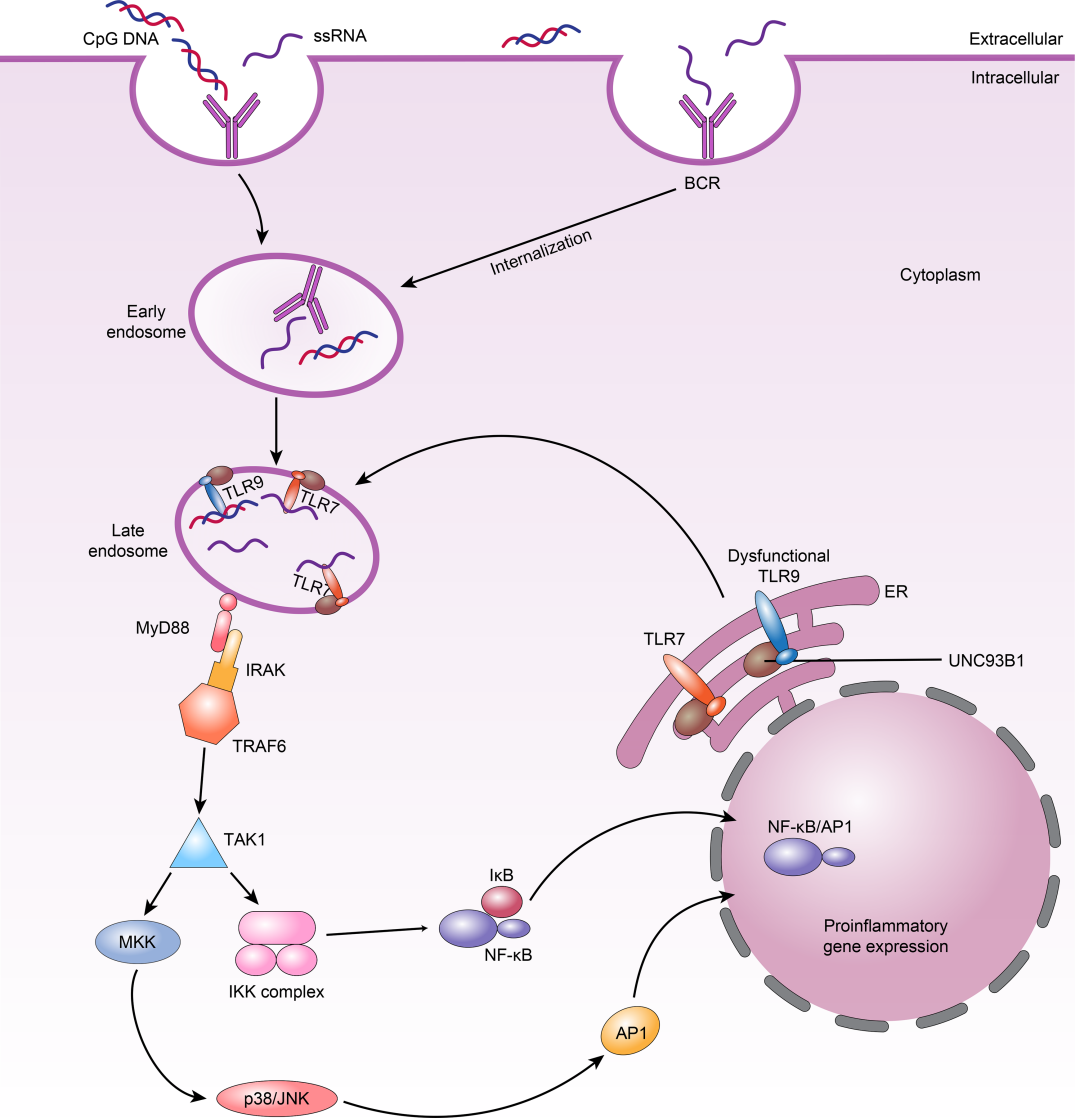
TLRs signalling in B cells and TLR7 and dysfunctional TLR9 compete for UNC93B1-dependent trafficking in SLE B cells. 1.When nucleic acid ligands are recognized by BCRs and internalized into B cells, they bind TLR7 or TLR9 in the late endosome; TLR7 or TLR9 then binds MyD88. MyD88 subsequently recruits the IRAK–TRAF6 complex, ultimately leading to activation of downstream NF-κB and AP1 signalling. 2.Dysfunctional TLR9 can lead to weakened competition of these TLRs for binding to UNC93B1 in endosomes, increasing TLR7 availability and resulting in higher TLR7 trafficking and responses, which manifest as autoimmune phenotypes.

All TLRs in B cells trigger similar signalling pathways that in turn activate NF-κB, MAPKs, extracellular signal-modulating kinase (ERK), p38, and Jun N-terminal kinase (JNK) (82). The common linker protein used by almost all TLRs is MyD88, which has death and toll interleukin 1 receptor (TIR) domains (83). When MyD88 binds to a TLR, it recruits members of the IL-1 receptor-associated kinase (IRAK) family, IRAK1 and IRAK4, by interacting with the death domains, thereby phosphorylating TNF receptor correlator 6 (TRAF6) (84). TRAF6 is phosphorylated by IRAKs and binds to Ubc13 and Uev1A to form a complex that activates a mitogen-activating protein kinase kinase (MAPKKK) called transforming growth factor β-activating kinase-1 (TAK-1). Activated TAK-1 phosphorylates MKKs, which are upstream kinases of p38 MAPKs and JNK (85). p38 is involved in many cellular physiological and pathological processes, including apoptosis, cellular stress, the cell cycle, and the inflammatory response (86). In addition, TAK-1 can activate the IκBα kinase (IKK) complex, consisting of the IKKα and IKKβ kinases and the hinge protein IKKγ (85). Phosphorylation of IκBα leads to its own degradation and the release of NF-κB (**Figure 2**). The NF-κB signalling pathway is critical in innate and adaptive immunity because it modulates the transcription of various immune-inflammatory genes, such as *TNF*, *IL-1*, and *IL-6*, resulting in chemotaxis, granulocyte and macrophage aggregation, and lymphocyte infiltration (84, 87, 88). Although the downstream pathways triggered by many TLRs are similar, TLR7 and TLR9 perform different functions in controlling the behavior of SLE B cells. We therefore hypothesize that in SLE, B-cell-specific signalling impacts the response of TLRs.

### 6.1 Interaction of BCRs and TLRs

B cells, in contrast to dendritic cells (DC), do not internalize external substances *via* microcytosis or endocytosis, so natural extracellular nucleic acids cannot directly contact TLR7 and TLR9 inside B cells. Instead, these TLRs are activated by BCR-antigen complexes that are delivered to late endosomes, where they trigger the costimulation of B cells (74, 89). This suggests that specific TLR-driven cell activation is associated with the expression of unique BCRs on B cells. In addition, mouse B cells that lack the BCR are unable to proliferate upon TLR agonist stimulation (90). Moreover, LAG-3^+^CD138^hi^ natural regulatory plasma cells, which produce IL-10 upon exposure to TLR agonists in mice, also grow in a BCR-dependent manner (91). These studies reaffirm that BCR activation facilitates endosomal TLR signalling in B cells, enabling these cells to react to TLR agonists (92).

### 6.2 Possible causes of impaired TLR9 function in SLE B cells

The molecular changes responsible for impaired TLR9 activity in SLE B cells are unclear. *TLR9* gene transcription is similar in B cells of both healthy individuals and SLE patients (15, 93). Hence, poor TLR9 responses in SLE B cells is unlikely due to decreased TLR9 expression (93). In SLE patients, TLR9 function is impaired in B cells but not in pDCs, indicating that B-cell-specific receptors may disturb the TLR9 response in SLE B cells. Several TLR9 actions in human B cells are mediated by CD19, which is expressed in B cells but not pDCs (15). CD19 is a transmembrane protein found on B cells that amplifies proximal BCR signalling by successively recruiting and activating LYN, PI3K, Bruton tyrosine kinase (BTK), and protein kinase B (AKT) (94). Likewise, B cells from individuals with one or two *CD19* allele variants exhibit lower or defective activation, respectively, in response to TLR9 agonist stimulation (94). This suggests that CD19 is indispensable for the control of TLR9 function in human B cells. Human SLE B cells have been shown to express less CD19 according to prior studies (95–97). In conjunction with this observation, Gies et al. (15) found that CD19 expression was lower on B cells from quiescent and active SLE patients. Furthermore, it has been demonstrated that the transitional B cells from individuals with SLE have abnormal phenotypes, including lower CD19 expression and impaired sensitivity to TLR9 stimuli (98). Previous studies have also shown that heterozygous and homozygous *CD19* gene mutations in B cells may be associated with the onset of SLE (99). However, such mutations are unlikely to frequently occur in SLE to alter the expression of CD19. Gies et al. (15) discovered that decreased CD19 expression on B cells from SLE patients normalized after 48 hours of *in vitro* culture, therefore, it is assumed that external factors are responsible for CD19 downregulation *in vivo*. Subsequent research has revealed that downregulation of CD19 on B cells is associated with the existence of anti-dsDNA antibodies, suggesting that immune complexes and apoptotic bodies containing DNA are involved in CD19 and TLR9 regulation in B cells in the bone marrow (BM) (98). By contrast, type I and II interferons, which are linked to the immunopathogenesis of SLE, are unable to modify CD19 (15). Primary Sjögren’s syndrome, a condition marked by the generation of anti-single-stranded RNA (ssRNA) antibodies but not anti-dsDNA antibodies, is characterized by ordinary CD19 expression on patient B cells (15). Consistent with this finding, DNA is found on the surface of B cells from SLE patients who are actively ill (but not on the surface of healthy donor cells) (100). However, whether CD19 downregulation in SLE B cells is a necessary factor for impaired TLR9 responses and leads to a break in immune tolerance in SLE B cells remains to be determined. Although both TLR7 and TLR9 activate downstream NF-κB and p38 MAPK signalling through MyD88 in B cells, data suggest that TLR7 does not promote B-cell death after proliferation in SLE *in vivo* and *in vitro*, but it does promote plasma cell differentiation (77). Interestingly, TLR7 function is preserved in SLE B cells, and the reasons for this effect are worth further investigations (98).

### 6.3 Effect of TLR9 dysfunction on B-cell differentiation

TLR9 dysfunction leads to a decrease in the differentiation of B cells into regulatory B cells (Bregs). Bregs are a class of immunomodulatory B cells that do not rely on the secretion of immunoglobulins (101). Upon stimulation by the TLR9 agonist CpG-DNA, these cells can secrete the cytokine IL-10 and effectively induce apoptosis of mouse and human effector T cells in an IL-10-dependent manner, which has a protective effect on the body under physiological conditions (57, 102). The TLR9 hyporesponsiveness of SLE B cells may underlie their inability to differentiate into IL-10-secreting Bregs (103). Diminished IL-10 secretion may then promote an increase in IFN-α production by pDCs from SLE patients. These pDCs are probably activated through their functional TLR9 by DNA-containing immune complexes, and this step may favour the progression of immature SLE B cells into plasmablasts, which frequently create autoreactive antibodies (15, 104). On the other hand, *TLR9*-deficient cells tend to differentiate into autoantibody-producing cells with expression of CD138 or other molecular markers of plasma cells *in vivo* and *in vitro* (77).

### 6.4 Opposing roles of TLR7 and TLR9 signaling in SLE B cells

Nündel et al. (77) found that even though *Tlr9*-deficient autoimmune disease-prone mice fail to generate an autoantibody response to dsDNA, the *Tlr9*-deficient cells continue to differentiate along the plasma cell lineage, and these mice develop more serious clinical disorders and have shorter lifespans. However, *Tlr7*-null and *Tlr7/9* double-null autoimmune disease-prone mice exhibit less severe disease. Furthermore, *Tlr9^−^/^−^* B cells predominantly become IgG autoantibody-producing cells *in vivo* when BCR/TLR7 is activated. Additionally, the functions of BCR/TLR7 may be at least partially limited by BCR/TLR9 activation (77). The dependence of protection on TLR7 elimination indicates that there may be direct interaction between TLR7 and TLR9 and that the negative influence of TLR7 on health is greater than the positive influence of TLR9. In fact, the transmembrane protein UNC93B1 plays a key role during the activation of TLR7 and TLR9 by trafficking them from the endoplasmic reticulum (ER) to late endosomes, where they communicate with their ligands (105). B cells from patients with active SLE have considerably higher UNC93B1 mRNA and protein levels than B cells from patients with inactive SLE and healthy controls (106). Further research found that knocking out *Unc93b1* extinguishes the response from TLR7 and TLR9 in B cells, and the levels of antibodies against Ro-52/60, La, DNA and cardiolipin are all apparently decreased in the KO mice (10, 107, 108). In addition, there is competition between TLR7 and TLR9 for UNC93B1 trafficking in B cells, in which TLR9 predominates due to its higher affinity for UNC93B1 (109). Thus, overactivation of TLR7 is regulated by UNC93B1 through balancing of TLR9 to TLR7 trafficking. Desnues et al. (110) showed that dysfunctional TLR9 can lead to weakened competition of these TLRs for binding to UNC93B1 in endosomes. This improves TLR7 availability and causes increased TLR7 trafficking and response, which can emerge as autoimmune symptoms (**Figure 2**). Significantly, it has also recently been shown that UNC93B1 can specifically suppress TLR7 signalling in B cells by recruiting syntenin-1 (111). Syntenin-1 binds to UNC93B1, causing TLR7 to move to intraluminal vesicles of multivesicular bodies and preventing TLR7 signalling (111).

However, impaired TLR9 does not seem to explain the exacerbation of the disease caused by *Tlr9*-KO, suggesting that the protective mechanisms of TLR9 in SLE involve non-classical pathways. Recent genetic studies of TLR9 functions in lupus uncovered complex regulatory and unclear proinflammatory functions of TLR9. Leibler et al. (17) constructed a murine model of lupus with TLR9 point mutations that either hinder ligand binding(*Tlr9* ^K51E^) or MyD88 signalling (*Tlr9* ^P915H^) and subsequently detected the effects of these mutations on the lupus phenotype. A scaffold protective role of TLR9 was identified in *Tlr9* ^K51E^ mice, which showed less severe disease than *Tlr9* ^−/−^control mice. This scaffolding role is dependent on the protein’s presence and supports the UNC93B1 hypothesis, which suggests that TLR7 endosomal localization should differ in *Tlr9* ^−/−^ and *Tlr9* ^WT^ cells (13, 112). However, there was not a marked difference in TLR7 endosomal or lysosomal localization, NF-κB signalling or TLR7-driven gene expression in mice with or without TLR9 (17). Despite the findings of Leibler et al. (17), studies have been unable to exclude a subtle impact of TLR9 expression on TLR7 activation and signalling, though this impact may not be a major reason why TLR9 restrains lupus. This calls into question the traditional conception that TLR9 only competitively restrains the signalling and expression of TLR7. Unexpectedly, compared with *Tlr9* ^K51E^ and *Tlr9* ^WT^ mice, *Tlr9* ^P915H^ mice exhibited greater protection, demonstrating that TLR9 also exerts a ligand-dependent but MyD88-independent control effect. Additionally, neither of the two abovementioned suppressive, MyD88-independent regulatory roles of TLR9 rely on the absence of anti-DNA antibodies, as neither of these mice produce anti-DNA antibodies, yet lupus is substantially ameliorated in *Tlr9* ^K51E^ and *Tlr9* ^P915H^ mice compared with *Tlr9* ^−/−^ mice (17). Furthermore, studies of triple chimaeras with WT, KO and mutant TLR9 haematopoietic cells in the bone marrow uncovered TLR9-MyD88-independent regulatory functions that were intrinsic to B cells and constrained the development of pathogenic ABCs and plasma cells (17). In addition, although Leibler et al. (17) were unable to exclude the possibility of regulatory anti-inflammatory functions of TLR9–MyD88 signalling, they did identify that the major impact of TLR9–MyD88 signalling is to accelerate disease through proinflammatory signalling. That TLR9 possesses unique regulatory functions and operates *via* proinflammatory and protective mechanisms that could explain why it differs from TLR7. In addition, BCR/TLR co-signalling, in which both ligands can be present in a single molecular complex, suggests that there are different roles for TLR7 and TLR9, and TLR9 may have unique roles in regulating BCR/TLR co-signals (16, 77). Ultimately, TLR9 plays a more complex role than TLR7 in SLE B cells, but further exploration is required.

## 7 Effects of other cells on the TLR7/TLR9 pathway in SLE B cells

pDCs are a dominant producer of IFN-I. The secretion of IFN-I by pDCs involves the TLR7/9-MyD88 signalling pathway (19). SLC15A4, which is a histidine transporter that controls lysosomal pH, is necessary for this process and impacts the production of autoantibodies (113–116). IFN-I signalling has a crucial role in SLE pathogenesis and specifically in the germinal centre response (117). By specifically upregulating TLR7 expression, pDC-derived IFN-I increases the TLR7 sensitivity of human naive B cells. The level of TLR9 expression, however, is unaltered (118). Soni et al. (119) experiments with mice showed that endosomal TLR7/TLR9 and IFN-I derived from pDCs promote the transformation of extrafollicular B-cells into short-lived antibody-forming cells (AFCs). Moreover, the optimal expansion of extrafollicular (ExFO) CD138^+^ B cells and ICOS^hi^ExFO-Th cells requires IFN-I (119). In addition, T cells participate in the proliferation and differentiation of SLE B cells. CD4^+^ effector T cells, including follicular helper T (Tfh) cells, are the main sources of TLR7-induced IFN-γ. Furthermore, IFN-γ signalling in SLE plays an indispensable role in TLR7-driven autoreactive B-cell development into antibody-secreting cells *via* germinal centre signalling (20, 21). The differentiation of SLE B cells into plasma cells is facilitated by IL-21 secreted by Tfh cells (18, 120).Additionally, T-Bet and CD11c expression in TLR-activated B cells is controlled by an interaction between IL-4, IL-21, and IFN-γ (121).. In addition, Soni et al. (119) showed that extrafollicular plasmablasts drive the autoantibody response in a T-cell-dependent manner. These autoantibodies bind to antigens to form immune complexes. On the one hand, immune complexes can cause damage to organs; on the other hand, they can be recognized by pDCs to produce IFN-I, thus forming a vicious cycle (119). Taken together, these findings suggest connections between pDCs, T cells, B cells, and endosomal TLRs (**Figure 1**).

## 8 Targeting TLR7/9 signaling to treat SLE

### 8.1 Targeting TLR7/9 signaling pathway-related molecules for SLE therapy

TLR7 is extremely important in the pathogenesis of SLE, so inhibitors of TLR7 may be a crucial component of SLE treatment. Indeed, some promising therapies for inhibiting signalling downstream of TLR7 are under clinical assessment. For example, several studies have demonstrated the safe and efficient use of an IRAK4 inhibitor in suppressing inflammatory gene expression and cytokine secretion in mouse and human peripheral blood mononuclear cells (122–124). Inhibitors of MyD88, IRAK1, TRAF6, BANK1 and TAK1, which are TLR7 signalling-related molecules, are also being developed (84, 125, 126). Notably, these approaches not only lack specificity for TLR7 over other endosomal TLRs but also may block the activation of various cell types in addition to B cells upon formation of SLE patient immune complexes or TLR7 ligand stimulation, including monocytes and pDCs. Therefore, it is essential to carefully assess the safety of these inhibitors. Recently, a new oral TLR7/8 inhibitor developed by Merck, KGaA in Germany known as enpatoran (M5049) was able to inhibit the production of IL-6 and IFN-α and is well tolerated in a dose-dependent manner in phase 1 clinical trials (127). It is expected that this drug can be used to treat SLE (128). Hydroxychloroquine (HCQ) plays an important role as an immunosuppressant in SLE and is widely used in the clinical treatment of autoimmune diseases. The mechanism includes direct binding to nucleic acids when they become concentrated in the endosome, which limits ligand binding to TLR7/9 (129). This leads to an impaired ability of pDCs from patients with SLE to produce IFN-α and TNF-α (130). However, given the therapeutic range and target population, HCQ may not be able to suppress immune storms once they progress because SLE is linked to multiple downstream pathways of TLR7/9 (131). In SLE, dysfunction of B-cell-intrinsic TLR9 prevents apoptosis of autoreactive B cells, so enhancing the TLR9 signal by upregulating CD19 may be a novel strategy for targeting TLR9 to treat SLE (13, 16, 98).

### 8.2 SLE treatment targeting trafficking mechanisms

In addition to UNC93B1 and syntenin-1, the MHCII-associated invariant chain has been shown to have a more significant impact in restricting TLR7 signalling by regulating its trafficking (132). In addition, studies have found that in mice, B cells with αv or β3 integrin deficiency display hyporesponsiveness to TLR signalling in the marginal zone and B-1a B-cell subsets and a reduction in GC formation upon immunization with TLR7 stimuli (133, 134). Hence, modulation of the function of UNC93B1, syntenin-1, the MHCII-associated invariant chain or αvβ3 integrin to affect TLR7 trafficking mechanisms in SLE B cells is an interesting strategy for treatment (111, 132, 135).

## 9 Conclusion

TLRs were originally identified as pattern recognition receptors and are now considered an important hub connecting innate and adaptive immunity. SLE is characterized by successive stimulation of the innate and adaptive immune systems by endogenous nucleic acids released by necrotic or apoptotic cells. TLR7 and TLR9, whose ligands are ssRNA and dsDNA, respectively, work as innate sensors for detecting viral infections. Endosomal TLR recognition of self-nucleic acids in B cells is considered a crucial step in the progression of SLE and leads to the formation of antinuclear antibodies. Both TLR7 and TLR9 mRNA are expressed to varying degrees in SLE B cells, but the two play divergent roles. TLR7 is a significant contributor to the pathogenesis of SLE in B cells, mediating GC responses and extrafollicular B-cell responses to promote the expansion of antibody-secreting cells and accelerate disease progression. However, B-cell-specific TLR9 appears to play a protective role in SLE. Moreover, the conventional views hold that impaired TLR9 function in SLE B cells leads to a breakdown of the balance of the TLR7 and TLR9 pathways, which manifests as enhanced TLR7 signalling, impaired apoptotic death after an initial proliferative burst, and increased conversion of B cells to plasma cells instead of Bregs, which eventually worsens the disease. However, recent research has found that B-cell-intrinsic TLR9 seems to have complex bidirectional regulatory roles in SLE, and its expression does not affect TLR7 signalling (17). These novel findings reveal unprecedented insights into TLR9 biology in environments beyond SLE. This unique dual regulatory role of TLR9 may be related to Toll–IL-1R (TIR) domain signalling from IL-1R family members, which have both proinflammatory and anti-inflammatory functions (136). Only 46% homology exists between the TIR domains of TLR7 and TLR9, explaining why these receptors have different functions. This may partly explain why TLR7 and TLR9 have different roles in SLE B cells. Ultimately, genetic assessment of the TIR domains in each protein and exploration at the biochemical level are needed to determine such differences.

Infection is known to be an important trigger exacerbating SLE, and TLR-induced innate immunity can kill pathogens to maintain homeostasis (137, 138). However, abnormal activation of TLR7 or impaired TLR9 function in B cells in patients with SLE leads to disruption of immune homeostasis and adaptive immune activation upon exposure to pathogens containing ssRNA and dsDNA. Therefore, therapy targeting B-cell TLR7/TLR9 has potential for SLE. Some drugs targeting TLR7 have been developed and applied in the clinic; however, drugs targeting B-cell TLR9 are still relatively lacking. We propose a method to enhance the function of TLR9 by upregulating the expression of CD19 in B cells, thereby restoring the postproliferative apoptosis pathway of TLR9. This is expected to prevent disruption of immune homeostasis in SLE patients upon infection with pathogens containing ssRNA and dsDNA, providing new ways to target B-cell TLR9 to treat SLE. Curiously, the expression of TLR9 in B cells of patients with SLE is upregulated and positively correlated with SLE disease activity. This seems to contradict the protective effects of TLR9 and may be the result of a feedback mechanism that upregulates the expression of TLR9 to induce protection. However, this hypothesis needs to be validated. Likewise, whether TLR9 expression and/or function differs between humans and mice remains unclear.

It should be noted that SLE is a disease involving multiple factors, so the study of SLE pathogenesis cannot be limited to B cells. A large number of studies have indicated that cytokines such as IFN and ILs derived from pDCs and T cells, the expression of CD19 and BCR, and the interaction of transporters such as UNC93B1 and SLC15A4 with TLRs operate in a complex network that controls autoreactive antibody responses to nucleic acid antigens and results in the complex pathogenesis and symptom heterogeneity of SLE. However, the interactions of these factors are still unclear and need to be further explored.

## Author contributions

LW drafted the manuscript. BZ revised and created some of the figures. XW, RL, HF, and LH read and revised the manuscript. ZZ and XM performed the literature search. C-QC participated in the revision of words and sentences. XS designed, revised and approved the final manuscript. All authors contributed to the article and approved the submitted version.

## Glossary

**Table:**

SLE	Systemic lupus erythematosus
Ags	Antigens
Ab	Antibody
TLR	Toll-like Receptor
BCR	B-cell receptors
IFN	Interferon
dsDNA	Double-stranded DNA
DC	Dendritic cells
pDCs	Plasmacytoid dendritic cells
GWAS	Genome-wide association studies
SNPs	Single-nucleotide polymorphisms
WT	Wild-type
GCs	Germinal centres
Spt-GCs	Spontaneous germinal centres
BLIMP-1	B lymphocyte-induced maturation protein 1
2’,3’-cGMP	2’,3’-Cyclic guanosine monophosphate
MyD88	Myeloid differentiation factor 88
TFs	Transcription factors
ABCs	Age-associated B cells
CpG	Cytosine–phosphate–guanine
LN	Lupus nephritis
IL	Interleukin
IRF4	IFN regulatory factor 4
PI3Ks	Phosphatidylinositol 3-kinases
NF-kB	Nuclear factor-kB
MAPK	Mitogen-activated protein kinase
BlyS	B-lymphocyte stimulator
ERK	Extracellular signal-modulating kinase
JNK	c-Jun N-terminal kinase
TNF	Tumour necrosis factor
IRAK	IL-1 receptor-associated kinase
TAK-1	Transforming growth factor β-activating kinase-1
IKK	IkBα kinase
TIR	Toll/interleukin 1 receptor
SYK	Spleen tyrosine kinase
BTK	Bruton tyrosine kinase
AKT	Protein kinase B
ssRNA	Single-stranded RNA
UNC93B1	Unc-93 homolog B1
JAK	Janus kinase
STAT	Signal transducer and activator of transcription
BM	Bone marrow
Bregs	Regulatory B cells
ER	Endoplasmic reticulum
SLC15A4	Solute carrier family 15 member 4
AFCs	Antibody-forming cells
ExFO	Extrafollicular
BANK1	B-cell scaffold protein with ankyrin repeats1
HCQ	Hydroxychloroquine
MHCII	MHC class II gene
